# Multidrug resistance, biofilm formation and detection of *bla*_CTX-M_ and *bla*_VIM_ genes in *E. coli* and *Salmonella* isolates from *chutney* served at the street-food stalls of Bharatpur, Nepal

**DOI:** 10.1016/j.heliyon.2023.e15739

**Published:** 2023-04-24

**Authors:** Sanjib Adhikari, Ramesh Sharma Regmi, Sanjeep Sapkota, Sujan Khadka, Nitendra Patel, Sandhya Gurung, Divya Thapa, Prabina Bhattarai, Prakriti Sapkota, Ranjana Devkota, Albert Ghimire, Komal Raj Rijal

**Affiliations:** a Department of Microbiology, Birendra Multiple Campus, Tribhuvan University, Bharatpur, Chitwan 44200, Nepal; b Central Department of Microbiology, Tribhuvan University, Kirtipur, Kathmandu 44618, Nepal

**Keywords:** *Chutney*, *E. coli*, ESBL, MBL, Biofilm, *Salmonella*

## Abstract

Antimicrobial resistance (AMR) amid the bacteria found in ready-to-eat foods is a grave concern today warranting an immediate intervention. The current study was undertaken to explore the status of AMR in *E. coli* and *Salmonella* species in ready-to-eat *Chutney* samples (n = 150) served at the street food stalls in Bharatpur, Nepal, with a major focus on detecting extended-spectrum β-lactamase (ESBL) and metallo β-lactamase (MBL) genes along with biofilm formation. Average viable counts, coliform counts, and *Salmonella Shigella* counts were 1.33 × 10^6^±141481.4, 1.83 × 10^5^±91303.6, and 1.24 × 10^5^±63933.19 respectively. Out of 150 samples, 41 (27.33%) harbored *E. coli*, of which 7 were *E. coli* O157:H7; whereas *Salmonella* spp. were found in 31 (20.67%) samples. Bacterial contamination of *Chutney* by *E. coli* and *Salmonella* and ESBL-production were both found significantly affected by different sources of water used, personal hygiene and literacy rate of the vendors as well as by the type of cleaning materials used to wash knives and chopping boards (*P* < 0.05). Antibiotic susceptibility testing revealed that imipenem was the most effective drug against both types of bacterial isolates. Additionally, 14 (45.16%) *Salmonella* isolates and 27 (65.85%) *E. coli* isolates were found to be multi-drug resistant (MDR). Total ESBL (*bla*_CTX-M_) producers reported were 4 (12.90%) *Salmonella* spp. and 9 (21.95%) *E. coli*. Only 1 (3.23%) *Salmonella* spp. and 2 (4.88%) *E. coli* isolates were *bla*_VIM_ gene carriers. Dissemination of knowledge of personal hygiene amongst the street vendors and consumer awareness regarding ready-to-eat foods are crucial factors that can be suggested to curtail the emergence and transmission of food-borne pathogens.

## 1 Introduction

Foodborne illness has been a common problem worldwide most probably due to changes in marketable food production, such as minimal processing, and changing consumer demands for ready-to-eat (RTE) meals [[1], [2], [3]]. Foodborne disease, particularly diarrhea, is a major public health concern caused by eating microbially contaminated foods [4]. Because of the rise in food-related illnesses, food safety issues have received more attention in recent years. Interventions aimed at the food production industry, food services, and consumers can all help to prevent foodborne diseases [5]. Street foods, according to World Health Organization (WHO), are food or beverage prepared or sold by hawkers in public places which are ready for consumption or can be consumed later without further processing. Street foods offer readily available, inexpensive, nutritious food and also offer a source of income for the vendors [6]. Despite the advantages in terms of inexpensiveness and convenience, concern for hygiene and safety is always raised [3]. Food prepared in small factories and sold by mobile vendors, food prepared at mobile vendors' homes and offered for public sale, and food prepared and sold on the street are the three main categories of street food shops [7].

*Chutney* is a nutritious condiment made from standard or seasonal fruits, vegetables, and herbs that are ground to a paste or a pulpy mash, and the required consistency is obtained by adding water or lime [8]. However, they undergo no processing, are consumed raw and therefore are the prominent source of foodborne illness [9]. The raw foods, particularly vegetables, salads, fruits, and sprouts, which are used as the raw material for the preparation of *Chutney*, are primarily responsible for the prevalence and growth of pathogens [10]. Some earlier studies have reported the presence of *Escherichia coli, Staphylococcus aureus, Bacillus* spp. and *Salmonella* spp. from *Chutney* [10,11].

Drug resistance is a never-ending process in nature that can be caused by a pre-existing factor in organisms or because of acquired factors [12]. Target gene mutation, drug inactivation, and decreased accumulation due to decreased permeability and/or increased efflux are all common causes of antibiotic resistance [13]. In recent days, the spread and emergence of the MDR bacteria in both clinical [14,15] as well as community settings [16,17], has become an intimidating concern. Likewise, food also serves as the vehicle for the transmission of several MDR [18] as well as ESBL-producing bacteria [[19], [20], [21]]. Bacteria can spread antimicrobial resistance genes to other bacteria via plasmid in minimally processed foods held under sub-lethal food preservation stresses such as high/low temperature, osmotic, and pH stress [22]. Multi-drug resistant bacterial isolates are becoming increasingly common in minimally processed foods [23].

Early and precise detection of ESBL and MBL-producing bacteria harbouring *bla*_CTX-M_ and *bla*_VIM_ gene is crucial because of the upsurge in the incidence, types, and dissemination of MBLs and ESBLs in the community settings. Carbapenem-intermediate or resistant outcomes from antibiotic susceptibility investigations suggest the notion of a possible MBL or ESBL production necessitating their confirmation phenotypically [14,24,25]. Biofilm-producing bacteria, according to infectious disease experts at the Centers for Disease Control and Prevention (CDC), could be responsible for 65% of all bacterial infections, compared to their free-floating counterparts [26]. Most importantly, production of biofilm, ESBL and MBL confer *E. coli* [14,27] as well as *Salmonella* [[28], [29], [30], [31]] with increased resistance towards an array of antibiotics; thereby, contributing to failures in the treatment of several infections.

In Bharatpur, most of the of street food handlers are unaware of good hygienic practices because they lack knowledge about hazardous consequences of contaminated foods and some of them are deliberately ignoring this issue and take it merely as a way to earn money by ignoring the health of the consumers. So far, there are very negligible efforts launched by Bharatpur Metropolitan City and other stakeholders in order to raise awareness on possible health issues caused by consuming unhygienic street foods. Considering these facts, the present study was carried out to investigate the incidence of two important food-pathogens: *Salmonella* and *E. coli* in street-vended *Chutney* with an emphasis on antimicrobial resistance, ESBL, MBL and biofilm production in the bacteria and exploring some pertinent factors that might have contributed to their emergence.

## 2 Methodology

### 2.1 Study site, design and sample size

**Fig. 1:**
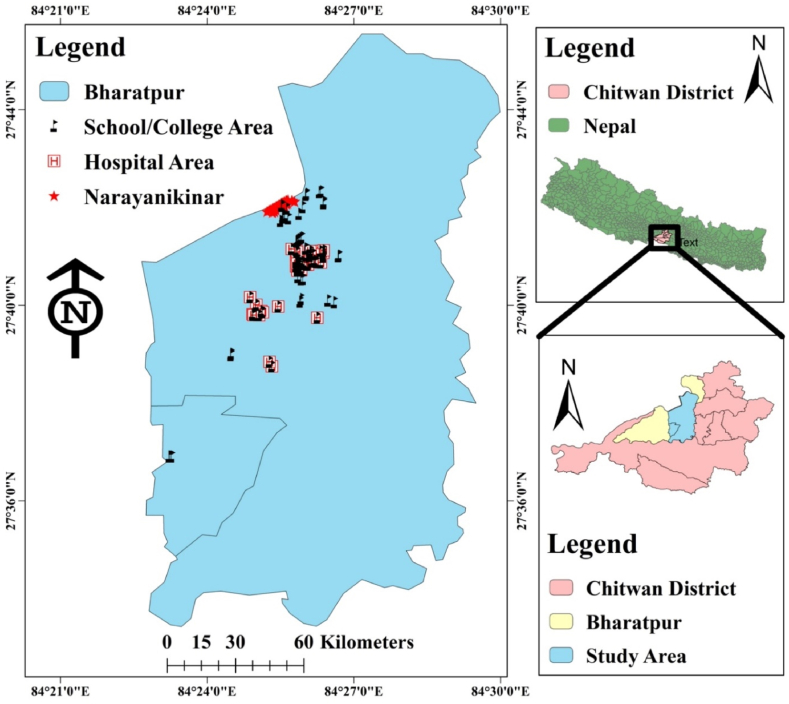
Location of the study area including sample collection sites in Bharatpur Metropolitan City, Nepal.

A descriptive cross-sectional study was carried out from September 2019 to March 2020 in Bharatpur Metropolitan City of Chitwan District, Bagmati Province, Nepal. *Chutney* samples (n = 150) were collected from 3 major crowded areas of Bharatpur city: Narayani kinar, hospital area and school/college area (50 samples from each area) (Fig. 1) using random sampling methods without repetition. The sample size was determined in accordance with the prevalence rate based on the previous study [21].

### 2.2 Sample collection and transportation

*Chutney* samples were collected in sterile zip-lock plastic bags from the street food stalls in Bharatpur city. The retrieved samples were placed in an icebox and transported aseptically to Birendra Multiple Campus's Microbiology laboratory within 1 h for further investigation.

### 2.3 Enumeration of bacteria from *chutney* sample

After transporting to the laboratory, *Chutney* (1 g) was immediately mixed with 9 mL of distilled water and serially diluted up to 10^−5^. Spread plate technique was performed using Plate count agar, Eosin Methylene Blue (EMB) agar and Xylose Lysine Deoxycholate (XLD) agar. The plates were aerobically incubated for 24 h at 37 °C and CFU/mL was determined [29].

### 2.4 Identification of the isolates

The suspected colonies of *E. coli* on EMB agar and *Salmonella* on XLD agar were further subcultured on Nutrient agar (NA). Bacterial isolates were identified by their cultural, morphological and biochemical tests [32]. *E. coli* isolates were streaked on MacConkey Sorbitol agar and incubated at 37 °C for 24 h for the preliminary identification of *E. coli* O157: H7, with colorless colonies suspected to be *E. coli* O157: H7. The slide agglutination test, which uses anti-O157 and flagellar H7 serum, was used to confirm *E. coli* O157: H7 (Defico, USA).

### 2.5 Antibiotic susceptibility test (AST) of the isolates

Conforming to Clinical and Laboratory Standards Institute (CLSI) recommendations, AST of *Salmonella* and *E. coli* isolates was performed using modified Kirby Bauer's disc diffusion method [33]. Altogether, 10 different commonly used antibiotics (co-trimoxazole (25 μg), ceftazidime (30 μg), chloramphenicol (30 μg), ciprofloxacin (5 μg), aztreonam (30 μg), ampicillin (25 μg), gentamicin (10 μg), imipenem (10 μg), amoxicillin (30 μg), nalidixic acid (300 μg)) obtained from Hi-Media, India, were used for testing. Organisms showing resistance to at least three antibiotics of different structural classes were considered MDR as documented elsewhere [21].

### 2.6 Phenotypic assessment of ESBL and MBL producers

The ceftazidime (30 μg) and cefotaxime (30 μg) discs were used to screen ESBL producing isolates. According to the Clinical and Laboratory Standards Institute, a prospective ESBL producer has a zone of inhibition (ZOI) of ≤22 mm for ceftazidime and ≤27 mm for cefotaxime. A combined disc test was used to confirm phenotypic confirmation of potential ESBL producers, as suggested by CLSI [33]. The validation of ESBL-producing isolates was accomplished using ceftazidime and cefotaxime alone and in combination with clavulanic acid (CA) (10 μg). An increment in ZOI of 5 mm for either antimicrobial drug tested in conjunction with CA as compared to its zone when tested alone was considered as ESBL production [21]. Imipenem-resistant isolates were chosen for further detection of MBL production using the disc potentiation method using imipenem (10 μg) and meropenem (10 μg) with and without EDTA (1 μg) [24]. Metallo beta-lactamase production was defined as a difference of ≥7 mm between the zones of inhibition of any of the carbapenem discs with or without chelating agents (EDTA) [34].

### 2.7 Detection of biofilm by microtiter plate method

Detection of biofilm formation was conducted by microtiter plate method as suggested by Stepanović et al. [35] and Kuinkel et al. [36] with slight adjustments. Briefly, a loopful of bacteria was inoculated in 10 mL of tryptic soya broth (TSB) supplemented with 1% glucose. After proper incubation at 37 °C for 24 h, the culture solution was diluted 100 times with freshly prepared TSB and 200 μL of diluted TSB was distributed to each well of a 96-welled microtiter plate. The plate was incubated at 37 °C for 24 h, the suspension was removed gently from the wells, washed with phosphate buffer saline (4 times), and fixation of biofilm attached to the walls of the plate was done with 2% sodium acetate. To visualize the biofilm, staining was done with 0.1% crystal violet while the extra stain was removed with de-ionized water followed by the drying of plates. To remove the dye from the cells, all wells were filled with 200 μL of 95% ethanol. The optical density (OD) of stained adherent biofilms was then read using a microtiter reader at 630 nm, and the results were categorized as weak, moderate, or strong biofilm producers. The optical density cut-off value (ODc) was calculated by arithmetically averaging the OD of the sterile TSB broth wells and adding a standard deviation of +2. Positive samples had an optical density greater than the cut-off value, while the negative samples had a lower optical density than the cut-off value. The following criteria were used to determine the capacity of biofilm production: no biofilm production (OD ≤ ODc), weak bioflim production (ODc < OD ≤ 2ODc), moderate biofilm production (2ODc < OD ≤ 4ODc), and strong biofilm production (OD > 4ODc) [35,36].

### 2.8 DNA extraction and amplification of *bla*_CTX-M_ and *bla*_VIM_ gene by PCR

All of the phenotypically confirmed ESBL and MBL producers were inoculated into 5 mL of Luria-Bertani broth (Hi-media, India) and incubated at 37 °C for 24 h. The plasmid DNA was extracted using the alkaline lysis technique [37]. After that, the DNA samples were suspended in 50 μL of TE buffer and kept at −20 °C. The genetic amplification was conducted in a 25 μL volume containing 12.5 μL master mix (Solis Biodyne, Estonia), 8.5 μL nuclease-free water, 3 μL of the plasmid DNA and 0.5 μL each of forward (*bla*_CTX-M_: 5′-TTT GCG ATG TGC AGT ACC AGT AA-3’; *bla*_VIM_: 5′-GAT GGT GTT TGG TCG CAT A-3′) and reverse (*bla*_CTX-M_: 5′-CTC CGC TGC CGG TTT TATC-3’; *bla*_VIM_: 5′-CGA ATG CGC AGC ACC AG-3′) primers (Macrogen, Korea) under the following optimal conditions: initial denaturation at 94 °C for 5 min, denaturation at 95 °C for 45 s of 35 cycles, annealing at 65 °C for 45 s of 35 cycles for *bla*_CTX-M_ and 56 °C for 45 s of 35 cycles for *bla*_VIM,_ extension at 72 °C for 30 s of 35 cycles for *bla*_CTX-M_ and 72 °C for 45 s of 35 cycles for *bla*_VIM_ and final extension at 72 °C for 10 min. The amplified PCR products were separated on a 1.5% agarose gel in 1× TAE buffer (0.04 Tris-acetate, 0.001 M EDTA, pH 8.0), dyed with ethidium bromide, and observed with a gel-doc system. The amplicon size for *bla*_CTX-M_ and *bla*_VIM_ were 560 bp [38] and 390 bp [39] respectively **(**Table 1**)**.

**Table 1:** Nucleotide sequence of the primer for the detection of *bla*_CTX-M_ and *bla*_VIM_ gene.

Gene	Primers (5′-3′)	Amplicon size (bp)	References
CTX-M	F: 5′-TTTGCGATGTGCAGTACCAGTAA-3′	560	[38]
	R: 5′-CTCCGCTGCCGGTTTTATC-3′		
VIM	F: 5′-GATGGTGTTTGGTCGCATA-3′	390	[39]
	R: 5′-CGAATGCGCAGCACCAG-3′		

### 2.9 Preservation of the *bla*_CTX-M_ and *bla*_VIM_ genes positive isolates

Bacterial isolates were preserved using the glycerol stock method with some modifications [40]. Overnight culture of bacteria in LB broth (0.5 mL) was added into 50% glycerol stock (0.5 mL) in Eppendorf tube with proper labeling. Culture-inoculated Eppendorf tubes were preserved in the refrigerator at −20 °C. Since subsequent freezing and thawing reduces the shelf life of the culture, while recovering, the frozen culture was scraped off with a sterile inoculating loop and directly transferred to culture media without letting the glycerol stock unthaw completely.

### 2.10 Quality control for test

Sterility and performance tests were performed on each batch of media and reagents. The control strains of *E. coli* ATCC 25922 and *Salmonella* Typhimurium ATCC 14028 were used to ensure quality control during the antibiotic susceptibility test. Media, antibiotics, and reagents were prepared, stored, and used according to the manufacturer's instructions for quality control. The antibiotic discs were kept in the refrigerator. For every PCR reaction, positive and negative controls were employed.

### 2.11 Data management and analysis

The SPSS software for windows (version 26) and R-programming statistical analysis tool were used to evaluate all the obtained data (version February 1, 5033, https://cran.r-project.org/). Chi-square test (χ^2^) and logistic regression were used to scrutinize the data, and ggplot2 (grammar of graphics, version 3.3.2) package was used to construct the plots. The degree of significant associations between the variables were presented as *P* < 0.001*** (highly significant), *P* < 0.01** (moderately significant) and *P* < 0.05* (significant). Likewise, Odds ratio (OR) and 95% confidence interval (CI) with both upper and lower limits were also calculated. Arc Geographic Information System (ArcGIS, version 10.2, https://www.arcgis.com/index.html) was used to locate the sampling points from the different sample collection areas.

## 3 Results

### 3.1 Distribution of *E. coli* and *Salmonella* in *Chutney*

**Fig. 2:**
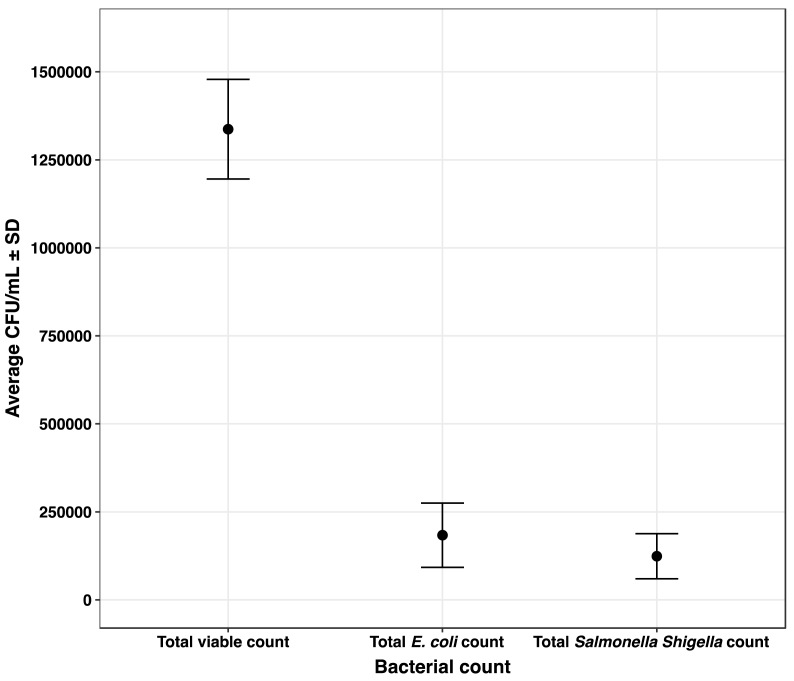
Average bacterial load from the collected samples.

**Fig. 3:**
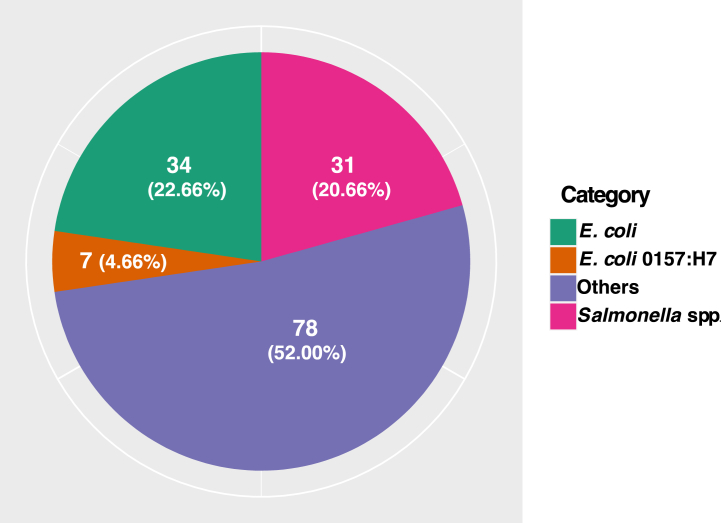
Distribution of bacterial isolates in street-vended *Chutney*.

*Chutney* samples were examined for the presence of viable bacteria. The average viable counts, coliform counts and *Salmonella Shigella* counts from the collected samples were 1.33 × 10^6^±141481.4, 1.83 × 10^5^±91303.6, 1.24 × 10^5^±63933.19 respectively (Fig. 2). *Salmonella* spp. was isolated from 31 (20.67%) samples, while *E. coli* was isolated from 41 (27.33%) samples. Seven (17.0%) of the *E. coli* isolates were *E. coli* O157: H7 (Fig. 3).

### 3.2 Bacterial contamination and associated factors

*Chutneys* served at School/College area were found to be more contaminated with *E. coli* (36.0%, *P* > 0.05, odds ratio (OR) = 1.78, 95% CI = 0.74–4.25) and *Salmonella* spp. (24.0%, *P* > 0.05, OR = 1.12, 95% CI = 0.44–2.84) than those from Narayani Kinar and Hospital area; however, the occurrence of these bacteria was not significantly associated with the area of sample collection. Lower incidence of *E. coli* (24.51%, *P* < 0.001, OR = 0.17, 95% CI = 0.08–3.73) and *Salmonella* (15.69%, *P* < 0.05, OR = 0.41, 95% CI = 0.18–0.92) as well as ESBL-producing *E. coli* (5.88%, *P* < 0.05, OR = 0.41, 95% CI = 0.18–0.92) was found in the *Chutney* prepared by the literate vendors as compared to those prepared by the illiterate ones. Similarly, lower prevalence of *E. coli* (6.90%, *P* < 0.05, OR = 0.16, 95% CI = 0.04–0.69) and *Salmonella* spp. (6.90%, *P* > 0.05, OR = 0.24, 95% CI = 0.05–1.05) was found in *Chutney* prepared by the gloved vendors than by the non-gloved vendors. Chopping boards and knives washed with water plus either with soap or detergent showed lower contamination of *E. coli* (16.07%, *P* < 0.001, OR = 0.13, 95% CI = 0.06–0.28), *Salmonella* spp. (14.29%, *P* < 0.01, OR = 0.25, 95% CI = 0.11–0.59), ESBL-producing *E. coli* (8.92%, *P* < 0.001, OR = 0.25, 95%, CI = 0.11–0.59) and ESBL-producing *Salmonella* (2.68%, *P* < 0.05, OR = 0.18, 95% CI = 0.04–0.80) in compared to those washed by water only. *Chutney* prepared by using underground water (open) documented higher occurrence of *E. coli* (64.29%, *P* < 0.01, OR = 6.26, 95% CI = 1.89–20.69), *Salmonella* spp. (57.14%, *P* < 0.001, OR = 7.61, 95% CI = 2.29–25.32), ESBL-producing *E. coli* (57.14%, *P* < 0.001, OR = 7.61, 95% CI = 2.29–25.32), as well as ESBL-producing *Salmonella* (21.43%, *P* < 0.01, OR = 12.54, 95% CI = 1.89–83.48) compared to the *Chutney* prepared by using municipal water and underground water (closed) (Table 2 and Table 3).

**Table 2:** Distribution of *Salmonella* and *E. coli* isolates among different attributes.

Attributes	No. of samples	Isolates					
		*E. coli*			*Salmonella* spp.		
		Growth rate	Odds ratio (95% CI)	p-value	Growth rate	Odds ratio (95% CI)	*P*-value
Areas							
Narayanikinar	50	12 (24.00%)	1	–	11 (22.00%)	1	–
Hospital area	50	11 (22.00%)	0.89 (0.35–2.26)	0.812	8 (16.00%)	0.68 (0.25–1.85)	0.446
School/college area	50	18 (36.00%)	1.78 (0.74–4.25)	0.192	12 (24.00%)	1.12 (0.44–2.84)	0.812
Literacy							
Literate	102	25 (24.51%)	0.17 (0.08–3.73)	0.000***	16 (15.69%)	0.41 (0.18–0.92)	0.030*
Illiterate	48	16 (33.33%)	1	–	15 (32.25%)	1	–
Personal Hygiene							
Gloved	29	2 (6.90%)	0.16 (0.04–0.69)	0.014*	2 (6.90%)	0.24 (0.05–1.05)	0.057
Non-gloved	121	39 (32.23%)	1	–	29 (23.97%)	1	–
Cleaning material							
Soap/Detergent	112	18 (16.07%)	0.13 (0.06–0.28)	0.000***	16 (14.29%)	0.25 (0.11–0.59)	0.001**
Water	38	23 (60.53%)	1	–	15 (39.47%)	1	–
Source of water used							
Municipal water	94	21 (22.34%)	1	–	14 (14.89%)	1	–
Ground water (closed)	42	11 (26.19%)	1.23 (0.53–2.86)	0.625	9 (21.43%)	1.56 (0.62–3.95)	0.349
Ground water (open)	14	9 (64.29%)	6.26 (1.89–20.69)	0.002 **	8 (57.14%)	7.61 (2.29–25.32)	0.000***

**Table 3:** Distribution of ESBL-producers among different attributes.

Attributes	No. of samples	Isolates					
		*E. coli*			*Salmonella* spp.		
		ESBL rate	Odds ratio (95% CI)	*P*-value	ESBL rate	Odds ratio (95% CI)	*P*-value
Narayani kinar	50	6 (12.00%)	1	–	2 (4.00%)	1	–
Hospital area	50	5 (10.00%)	0.68 (0.25–1.85)	0.446	2 (4.00%)	1 (0.14–7.39)	1.00
School/college area	50	10 (20.00%)	1.12 (0.44–2.84)	0.812	4 (8.00%)	2.09 (0.37–11.95)	0.409
Literacy							
Literate	102	6 (5.88%)	0.41 (0.18–0.92)	0.031*	4 (3.92%)	0.45 (0.11–1.88)	0.273
Illiterate	48	15 (31.25%)	1	–	4 (8.33%)	1	–
Personal Hygiene							
Gloved	29	1 (3.44%)	0.41 (0.18–0.92)	0.057	1 (3.45%)	0.58 (0.07–4.92)	0.619
Non-gloved	121	20 (16.52%)	1	–	7 (5.79%)	1	–
Cleaning material							
Soap/Detergent	112	10 (8.92%)	0.25 (0.11–0.59)	0.001**	3 (2.68%)	0.18 (0.04–0.80)	0.024*
Water	38	11 (28.94%)	1	–	5 (13.16%)	1	–
Source of water used							
Municipal water	94	11 (11.70%)	1	–	2 (2.13%)	1	–
Ground water (closed)	42	2 (4.76%)	1.56 (0.62–3.95)	0.349	3 (7.14%)	3.54 (0.57–22.01)	0.175
Ground water (open)	14	8 (57.14%)	7.61 (2.29–25.32)	0.000***	3 (21.43%)	12.54 (1.89–83.48)	0.008**

### 3.3 Antibiotic susceptibility pattern (AST) of the isolates

**Fig. 4:**
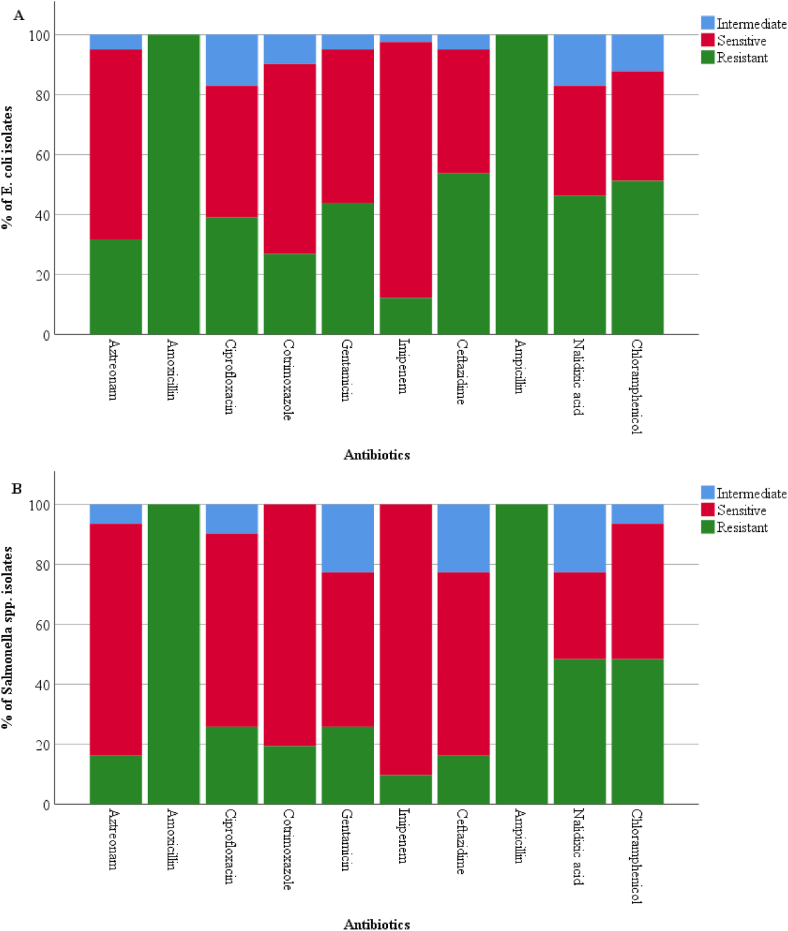
AST pattern of *E. coli* (A) and *Salmonella* spp. (B) isolated from *Chutney*.

Imipenem was the most efficient antibiotic as 87.80% *E. coli* and 90.32% *Salmonella* spp. isolates were sensitive to it. Co-trimoxazole, ciprofloxacin and gentamicin were the drugs which were effective at a fair rate against both the isolates. Amoxicillin and ampicillin were the least effective drugs against both isolates as *Salmonella* and *E. coli* isolates completely resisted these drugs (Fig. 4A and B). Among 41 *E. coli* isolates*,* 27 (65.85%) isolates and 14 (45.16%) *Salmonella* spp. among 31 isolates were MDR.

### 3.4 Distribution of ESBL- and MBL-producing *E. coli* and *salmonella* isolates

**Fig. 5:**
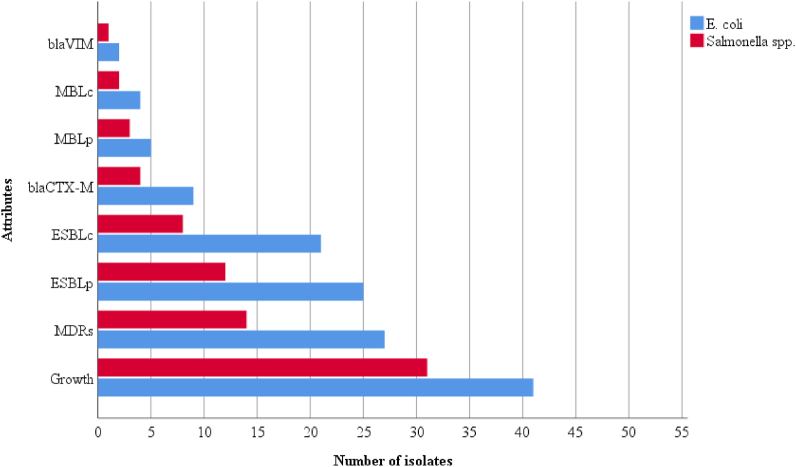
Distribution of ESBL, MDR and MBL isolates. Note: (p); Presumptive, (c); Confirmatory.

**Fig. 6:**
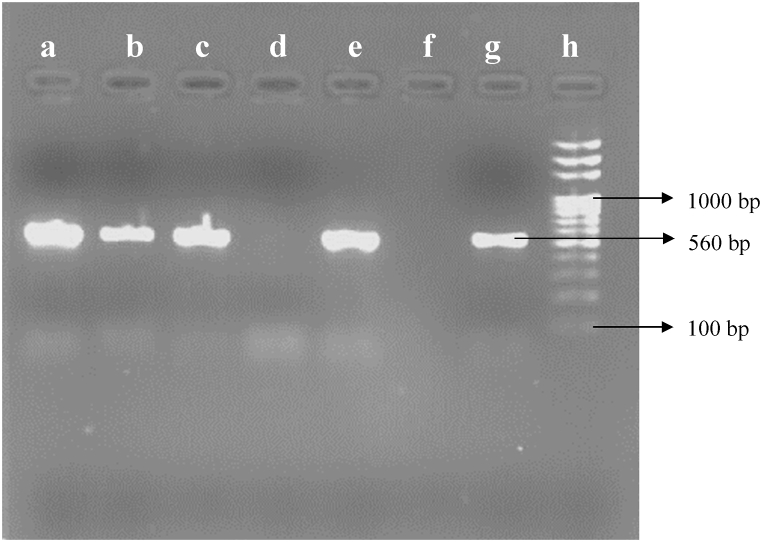
Amplification of *bla*_CTX-M_ gene. Lane a, b, c, e; *bla*_CTX-M_ positive isolates, Lane d; *bla*_CTX-M_ negative isolates, Lane f; negative control (nuclease-free water), Lane g; positive control, Lane h; 100bp ladder.

**Fig. 7:**
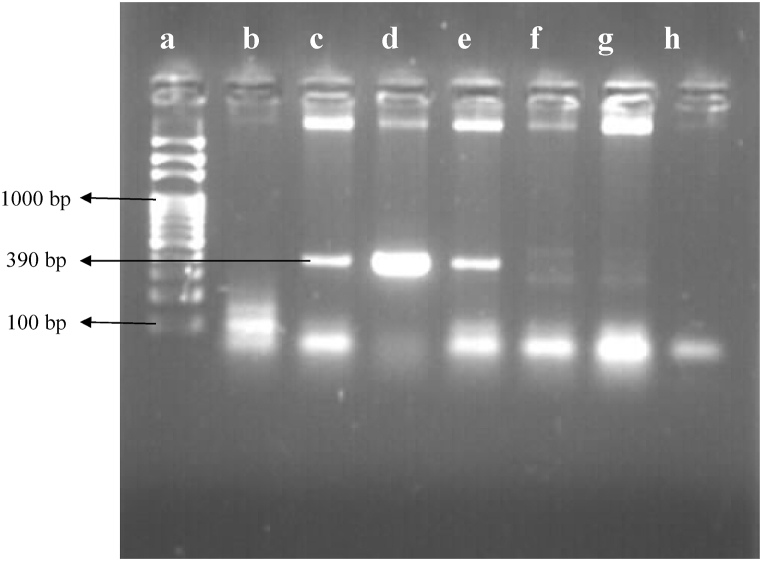
Amplification of *bla*_VIM_ gene. Lane a; 100bp ladder, lane b; negative control (nuclease-free water), Lane c; positive control, Lane d, e; *bla*_VIM_ positive isolates, Lane f, g, h; *bla*_VIM_ negative isolates.

The ESBL-screening test was positive in 37 out of the 72 isolates. The combined disk-diffusion test revealed that 21 isolates of *E. coli* and 8 isolates of *Salmonella* spp. were ESBL producers. Nine (21.95%) *E. coli* and 4 (12.90%) *Salmonella* isolates documenting a total of 13 (18.06%) possessed *bla*_CTX-M_ gene. Only 5 isolates of *E. coli* and 3 isolates of *Salmonella* spp. were found to be MBL-screening positive, out of which 4 isolates of *E. coli* and 2 isolates of *Salmonella* were confirmed as MBL producers. In addition, *bla*_VIM_ genes were detected in only 2 (4.88%) *E. coli* and 1 (3.23%) *Salmonella* isolates. No isolates harbouring both the genes were reported (Fig. 5). PCR amplification of *bla*_CTX-M_ and *bla*_VIM_ gene is shown in Fig. 6, Fig. 7.

### 3.5 Association of biofilm production with MDR

**Fig. 8:**
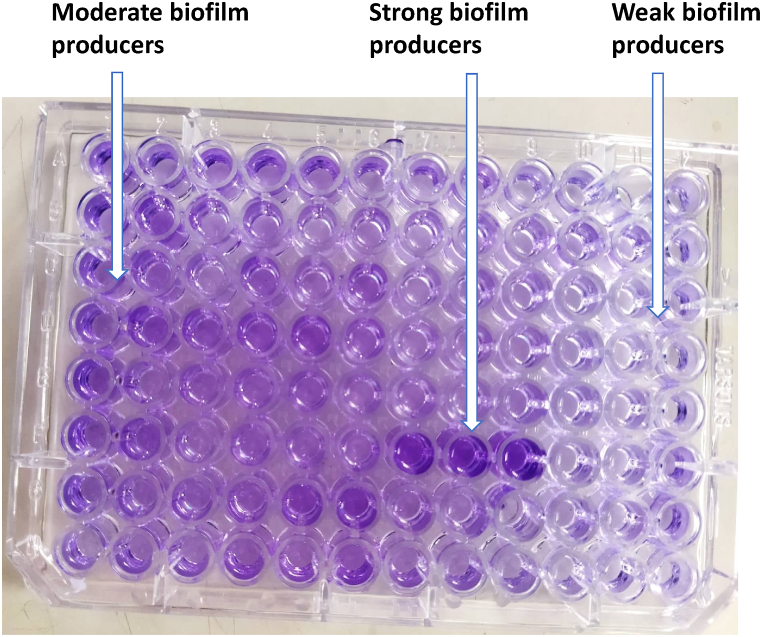
Microtiter plate assay indicating moderate, strong, and weak biofilm formation.

In the current study, overall, 30 (41.67%) isolates were biofilm producers, of which 14 (46.67%) isolates were weak, 9 (30%) were moderate and remaining 7 (23.33%) were strong producers (Fig. 8). In addition, 19 (46.34%) *E. coli* isolates were biofilm producers among which 17 isolates were MDR. Similarly, of the 11 (35.48%) *Salmonella* spp., 6 were MDR (Table 4).

**Table 4:** Association between biofilm production and MDR isolates.

Isolates	n	MDR isolates	Biofilm producers	MDRs among biofilm producers	p-value
*E. coli*	41	27 (65.85%)	19 (46.34%)	17 (89.47%)	0.219
*Salmonella* spp.	31	14 (45.16%)	11 (35.48%)	6 (54.55%)	
Total	72	41 (56.94%)	30 (41.67%)	23 (76.67%)	

### 3.6 Association between the presence of *bla*_CTX-M_ and *bla*_VIM_ genes with multidrug resistance, ESBL and MBL production

Amongst 41 *E. coli* isolates, the rate of MDR, ESBL and MBL production was observed higher (77.78%, 100.00% and 22.22% respectively) in the isolates which bore *bla*_CTX-M_ gene as compared to the isolates which lacked the *bla*_CTX-M_ gene (62.5%, 37.5%, 62.5% respectively). All *Salmonella* isolates carrying the *bla*_CTX-M_ gene were MDR and ESBL producers, while 25% of them were found to produce MBL. Likewise, all bacterial isolates harbouring *bla*_VIM_ gene were reported to be MBL producers. In contrast, significant association was observed between *bla*_CTX-M_ possession, MDR status and ESBL production in case of *Salmonella* spp.; whereas only between *bla*_CTX-M_ possession and *ESBL* production in case of *E. coli* (*P* < 0.05) (Table 5).

**Table 5:** Association between presence of *bla*_CTX-M_ and *bla*_VIM_ genes with multidrug resistance (MDRs), ESBL production (ESBLp) and MBL production (MBLp) among *E. coli* and *Salmonella* spp. Note: # either all or no resistance was detected, hence, *P*-value could not be analyzed; p: Phenotypically confirmed, *: Significant at 5% level of significance.

Bacteria	Genes		MDRs %	*P*-value	ESBLp %	*P*-value	MBLp%	*P*-value
*E. coli*	*bla*_CTX-M_	Positive	77.78%	0.393	100%	0.0009*	22.22%	0.1536
			(7/9)		(9/9)		(2/9)	
		Negative	62.50% (20/32)		37.50%		62.50%	
					(12/32)		(2/32)	
*Salmonella*	*bla*_CTX-M_	Positive	100%	0.018*	100%	0.0003*	25%	0.1057
			(4/4)		(4/4)		(1/4)	
		Negative	37.03% (10/27)		14.82%		3.70%	
					(4/27)		(1/27)	
*E. coli*	*bla*_VIM_	Positive	50%	0.6278	0%	0.1373	100%	#
			(1/2)		(0/2)		(2/2)	
		Negative	66.67% (26/39)		53.85%		5.13%	
					(21/39)		(2/39)	
*Salmonella*	*bla*_VIM_	Positive	0%	0.3563	0%	0.5488	100%	#
			(0/1)		(0/1)		(1/1)	
		Negative	46.67% (14/30)		26.67%		3.33%	
					(8/30)		(1/30)	

## 4 Discussion

In developing countries like Nepal, street foods are probably one of the prominent sources of food-borne illnesses, yet they are preferred and consumed by a large chunk of people. The present study investigated the bacterial quality of *Chutney* served at different street-vended shops with a major focus on *Salmonella* and *E. coli*.

A total of 150 *Chutneys* sold at the street food stalls in different areas of Bharatpur city were subjected to bacterial investigation. *Chutney* samples had average CFU counts of 1.33 × 10^6^±141481.4, 1.83 × 10^5^±91303.6, and 1.24 × 10^5^±63933.19 for total viable growth, total coliform growth and total *Salmonella Shigella* growth respectively. These counts are higher than those reported in a study conducted by Eromo in Ethiopia, where the count was 1.7 × 10^5^ in *Chutney* (Awaze) samples [41]. Another Ethiopian study reported that the CFU in street food samples was 6.64 × 10^4^ [42]. In contrary, the mean CFU count of this study is lower than the study done in Gondar where it was 5.1 × 10^7^ [43]. There is a variation in the rate of bacterial contamination of *Chutney* in different regions owing to many factors such as the environmental conditions which determine the bacterial growth and also the different types of ingredients (raw vegetables) and water used at different places to prepare *Chutney* [5]. Out of 150 samples, 27.33% samples were contaminated with *E. coli*. This rate is lower than some similar studies done in India and Bangladesh, which recorded 55.00% and 46.00% samples contaminated with *E. coli* respectively [44,45]*.* The incidence of *Salmonella* spp. reported in this study was 20.67%. A similar study in Deharadun city recorded 30.00% of *Chutney* contaminated with *Salmonella* spp. [44]. Though the rate of contamination is different, the nature of the contamination is similar as most of the studies in street foods have recorded a higher prevalence of *E. coli* than *Salmonella* isolates [21,44,45]. In the present study, *E. coli* O157: H7 have been recovered in 4.67% of *Chutney* samples. A study done in green salads served at hotels and restaurants of the same city, Bharatpur, showed 1.70% prevalence of *E. coli* O157: H7 [21]. *E. coli* O157: H7 contamination is more likely in vegetables grown in soil fertilized with animal manure [46].

Most of the *Chutney* samples from street food stalls located near school/college were found contaminated by *E. coli* (36.00%) and *Salmonella* spp. (24.00%) as compared to the food stalls at Narayani kinar and Hospital area, though it was not found statistically significant (*P* > 0.05). This can be ascribed to the fact that school/college areas are more crowded, which increases the chance of bacterial contamination leading to the food borne illnesses. The literacy rate of the chefs/vendors is an important factor that defines the extent to which the foods are contaminated. *Chutney* prepared by illiterate vendors had a higher incidence of *E. coli* (33.33%) (*P* < 0.001) and *Salmonella* spp. (31.25%) (*P* < 0.05). Vendors who are illiterate are unaware of health and hygiene issues, and they may cross-contaminate the *Chutney* during the preparation process. Vendors' cleanliness habits may have a direct impact on the dissemination of *E. coli* and *Salmonella*. *Chutney* prepared by non-gloved vendors accounted for a higher growth rate of *E. coli* (32.23%) (*P* < 0.05) and *Salmonella* spp. (23.97%). This finding echoes with another study which documented that non-gloved vendors served highly contaminated ready-to-eat foods compared to gloved vendors [18]. Foodborne pathogens such as *Salmonella* and *E. coli* O157: H7 are easily transmitted by contaminated hands [47]. Non-gloved vendors/persons may carry dirt and dust particles in their hands, which may contain a larger number of germs that can contaminate the *Chutney* while preparing. The cleaning trend of chopping boards and knives affects the frequency of *Salmonella* in *Chutney* [48]. A higher growth rate of *E. coli* (60.53%) and *Salmonella* spp. (39.47%) was documented in the *Chutney* sold by the vendors who use water only to wash chopping board and knives (*P* < 0.05). The finding is in accordance with a previous study conducted with green salads served at hotels and restaurants in Bharatpur city [21]. Disinfection methods can lessen bacterial burdens on non-living surfaces, resulting in reduced coliform counts [49]. *Chutney* prepared by using underground water (open) accounted for higher occurrence of *E. coli* (64.29%) and *Salmonella* spp. (57.14%) in the present study. Municipal water is supplied only after treating with disinfectant which kills the bacteria present in it, and underground water (closed) has very little chance of being contaminated with microbes; whereas there is always a greater chance of contamination of open sources of water. Various factors that might contribute to the contamination of open water sources (wells) are-proximity of latrine to the wells, haphazard use of wells and waste management pits near the water sources [50]. Consumers’ awareness on food safety is very important in order to curtail the incidences of foodborne illnesses and infections.

On performing antimicrobial susceptibility testing, imipenem and co-trimoxazole were found to be the most effective antibiotics against both the bacterial pathogens*.* A study performed in the same city revealed that co-trimoxazole was the most effective drug with 93.02% and 97.00% sensitivity against *E. coli* and *Salmonella* spp. detected from green salads respectively [21]. But another study done in street-vended *Panipuri* of the same city noted a lower sensitivity rates (46.67% and 42.85%) of co-trimoxazole against these two bacteria [18]. A similar study done on street foods in Ethiopia reported gentamycin (93.33%) and chloramphenicol (86.67%) sensitivity rates higher than our study [42]*.* Another Ethiopian study reported 88.90% resistance to chloramphenicol by *Salmonella* spp. which is higher than our study (48.39%) [41]. Amoxicillin and ampicillin were found to be the most ineffective drugs as all the isolates of *E. coli* and *Salmonella* spp. resisted these drugs. Some other studies on street foods also reported ampicillin as the least effective drug [41,42]. However, a study performed by Khadka et al. showed only 20.00% isolates of *E. coli* and 17.85% of *Salmonella* spp. resisted this drug [18]. The low efficacy of these medications could be owing to simple hydrolysis of the β-lactam ring by *Salmonella* and *E. coli*, as well as the widespread use of β-lactam antibiotics due to their low cost, leading to the development of resistance in most bacteria [51,52].

In our study, 65.85% isolates of *E. coli* and 45.16% *Salmonella* isolates were MDR. The rate of MDR isolates in this study is lower than some other studies done in vegetables and fruits [53,54]. In contrast, a study performed on street-vended *Panipuri* in the same city has reported only 36.70% *E. coli* and 33.33% *Salmonella* spp. as MDR. The problem of antibiotic resistance, which can be ascribed to irrational use of antibiotic, is increasing enormously day by day and is threatening the medical fraternity. The prevalence of ESBL-producing (*bla*_CTX-M_ gene) *E. coli* and *Salmonella* spp. was 21.95% and 12.90% respectively in our study. A study done on retail foods in China reported 9.4% ESBL-producing Enterobacteriaceae [20]. Meanwhile, a study conducted by Raphael et al. [55] observed an ESBL incidence rate of only 2.3% among bacterial isolates from spinach sold at street vended shop; whereas another study performed in Bharatpur city recorded only 2.3% *Salmonella* isolates and 1.85% *E. coli* isolates as ESBL producers [21]. Little is known about the transfer of ESBL-producing bacteria in food, but the endogenous fecal flora of animal origin can certainly spread across the food chain and contaminate water [19]. Also, horizontal transfer of ESBL producers from humans to foods may occur due to the sheer negligence of food handlers [56]. This study recorded 4.88% and 3.23% isolates of *E. coli* and *Salmonella* spp. carrying *bla*_VIM_ gene respectively. Iseppi et al. reported only 1.28% of isolates carrying *bla*_VIM_ gene in ready-to-eat foods [25]. Following the emergence of ESBL-producing bacteria in the 2000s, usage of carbapenem antibiotics increased drastically resulting in the increased number of beta-lactamases-producing bacteria that can even hydrolyze carbapenems [57].

Of the 41 isolates of *E. coli*, 19 (46.34%) were biofilm producer among which 17 isolates were MDR, whilst 11 (35.48%) isolates were biofilm producers in case of *Salmonella* spp., of which 6 were MDR. Few years back, Behzadi et al. also reported that production of biofilm resulted in resistance to multiple classes of antibiotics [58] and not only in Enterobacteriaceae family, there has been report of similar trends in *Pseudomonas* spp. and *Acinetobacter* spp. as well [59]. Present study showed that the prevalence of MDR, ESBL and MBL production is pretty higher among the *E. coli* isolates possessing *bla*_CTX-M_ gene as compared to the isolates which lacked *bla*_CTX-M_ gene (77.78%, 100.00%, 22.22% vs. 62.50%, 37.50%, 62.50%) for MDR, ESBL and MBL respectively. All bacterial isolates harbouring *bla*_VIM_ gene were reported to be MBL producers in the present study. Higher antimicrobial resistances are observed in *E. coli* and *Salmonella* harbouring *bla*_CTX-M_ and *bla*_VIM_ genes and are associated with the spread of clonal lineages as suggested by the previous findings [14,[60], [61], [62]].

## 5 Conclusion

The results of the study hint that a poor quality of *Chutney* is being served to the consumers eating street-vended foods. Use of untreated water from open wells, unawareness about personal hygiene and improper cleaning of the utensils all have contributed to *Chutney* contamination by *Salmonella* and *E. coli*. The emergence of ESBL, MBL and biofilm-producing isolates in ready-to-eat *Chutney* is worrisome as it might lead to serious health consequences. Concerned authorities should act seriously and quickly to prevent outbreaks of food-borne illnesses and the spread of AMR in the metropolis.

### 5.1 Strengths and limitations

To the best of our knowledge, this is the first attempt exploring both phenotypic as well as the genotypic studies of ESBL, MBL and biofilm-producing *E. coli* and *Salmonella* from the *Chutney* samples sold in street-vended shops in Nepal. The results of the study might be helpful to the stakeholders to formulate policies and conduct programs related to food safety and consumer awareness. On the downside, the current work is confined to investigating only two bacterial pathogens (*E. coli* and *Salmonella*) and also to the molecular identification of only two genes. Furthermore, the potential sources of contamination of the *Chutney* were not identified. Future studies are recommended to address these limitations.

## Contributions

SA, SK, SS and RSR conceived and designed the experiment. NP, SG, PB, PS, DT, RD, RSR carried out the laboratory works. SK, RSR and SS wrote the manuscript. SK and SA analyzed the data. SA, SK, AG, SS and KRR supervised the work. The final manuscript was reviewed and approved by all contributors.

## Data availability

The datasets generated during and/or analyzed during the current study are available from the corresponding author on reasonable request.

## Ethics approval and consent to participate

Not needed.

## Consent of publication

Not applicable.

## Declaration of competing interest

The authors declare that they have no known competing financial interests or personal relationships that could have appeared to influence the work reported in this paper.

## References

[1] Mead P.S., Slutsker L., Dietz V., McCaig L.F., Bresee J.S., et al Shapiro C. (1999). Food-related illness and death in the United States. Emerging infectious diseases. Emerg. Infect. Dis..

[2] Nguz K. (2007). Assessing food safety system in sub-Saharan countries: an overview of key issues. Food Conrol.

[3] Kaneko K.-I., Hayashidani H., Ohtomo Y., Kosuge J., Kato M. (1996). Takahashi, Bacterial contamination of ready-to-eat foods and fresh products in retail shops and food factories. J. Food Protect..

[4] Taylor C.E., Greenough W.B. (1989). Control of diarrheal diseases. Annu. Rev. Publ. Health.

[5] Adams M., Moss M. (2007).

[6] Imathiu S. (2017). Street vended foods: potential for improving food and nutrition security or a risk factor for foodborne diseases in developing countries?. Curr. Res. Nutr. Food Sci..

[7] Chakravarty I., Canet C. (1996). Street foods in Calcutta. Food Nutr. Agric..

[8] Barrett D.M., Beaulieu J.C., Shewfelt R. (2010). Color, flavor, texture, and nutritional quality of fresh-cut fruits and vegetables: desirable levels, instrumental and sensory measurement, and the effects of processing. Crit. Rev. Food Sci. Nutr..

[9] Viswanathan P., Kaur R. (2001). Prevalence and growth of pathogens on salad vegetables, fruits and sprouts. Int. J. Hyg Environ. Health.

[10] Ghosh M., Bansal S., Ganguli A. (2004). Prevalence and survival of S. aureus in street vended Indian green chutneys. Indian J. Med. Microbiol..

[11] Wadhai V.S., Khobragade K.D. (2012). Evaluation of microbiological safety of Indian chutneys: a case study of Chandrapur City, India. Sci Res Rep.

[12] Manikandan S., Ganesapandian S., Singh Manoj, Kumaraguru A.K. (2011). Emerging of multidrug resistance human pathogens from urinary tract infections. Curr. Res. Bacteriol..

[13] White D.G., Zhao S., McDermott P.F., Ayers S., Gaines S., Friedman S., DebRoy C. (2002). Characterization of antimicrobial resistance among Escherichia coli O111 isolates of animal and human origin. Microb. Drug Resist..

[14] Manyahi J., Kibwana U., Mgimba E., Majigo M. (2020). Multi-drug resistant bacteria predict mortality in bloodstream infection in a tertiary setting in Tanzania. PLoS One.

[15] Adhikari S., Khadka S., Sapkota S., Rana J.C., Khanal S., Neupane A., Sharma B. (2019). Prevalence and antibiograms of uropathogens from the suspected cases of urinary tract infections in Bharatpur hospital, Nepal. J. Coll. Med. Sci..

[16] Huang Y.S., Lai L.C., Chen Y.A., Lin K.Y., Chou Y.H., Chen H.C., Wang S.S., Wang J.T., Chang S.C. (2020). Colonization with multidrug-resistant organisms among healthy adults in the community setting: prevalence, risk factors, and composition of gut microbiome. Front. Microbiol..

[17] Sapkota S., Khadka S., Adhikari S., Parajuli A., Kandel H., Regmi R.S. (2020). Microbial diversity and antibiotic susceptibility pattern of bacteria associated with motorcycle helmets. Internet J. Microbiol..

[18] Khadka S., Adhikari S., Rai T., Ghimire U., Parajuli A. (2018). Bacterial contamination and risk factors associated with street-vended Panipuri sold in Bharatpur, Nepal. Int. J. Food Res..

[19] Tham J., Walder M., Melander E., Odenholt I. (2012). Prevalence of extended-spectrum beta-lactamase-producing bacteria in food, Infect. Drug Res..

[20] Ye Q., Wu Q., Zhang S., Zhang J., Yang G., Wang J., Xue L., Chen M. (2018). Characterization of extended-spectrum β-lactamase-producing Enterobacteriaceae from retail food in China. Front. Microbiol..

[21] Sapkota S., Adhikari S., Pandey A., Khadka S., Adhikari M., Kandel H., Pathak S., Pandey A. (2019). Multi-drug resistant extended-spectrum beta-lactamase producing E. coli and Salmonella on raw vegetable salads served at hotels and restaurants in Bharatpur, Nepal. BMC Res. Notes.

[22] Hirt H., Schlievert P.M., Dunny G.M. (2002). In vivo induction of virulence and antibiotic resistance transfer in Enterococcus faecalis mediated by the sex pheromone-sensing system of pCF10. Infect. Immun..

[23] Cocconcelli P.S., Cattivelli D., Gazzola S. (2003). Gene transfer of vancomycin and tetracycline resistances among Enterococcus faecalis during cheese and sausage fermentations. Int. J. Food Microbiol..

[24] Chika E., Malachy U., Ifeanyichukwu I., Peter E., Thaddeus G., Charles E. (2014). Phenotypic detection of metallo - β - lactamase (MBL) enzyme in enugu. Southeast Nigeria.

[25] Iseppi R., De Niederhaüsern S., Bondi M., Messi P., Sabia C. (2018). Extended-spectrum β-lactamase, AmpC, and MBL-producing gram-negative bacteria on fresh vegetables and ready-to-eat salads sold in local markets. Microb. Drug Resist..

[26] Potera C. (1999). Forging a link between biofilms and disease. Science.

[27] Dumaru R., Baral R., Shrestha L.B. (2019). Study of biofilm formation and antibiotic resistance pattern of gram-negative Bacilli among the clinical isolates at BPKIHS, Dharan. BMC Res. Notes.

[28] González J.F., Alberts H., Lee J., Doolittle L., Gunn J.S. (2018). Biofilm Formation protects Salmonella from the antibiotic ciprofloxacin in vitro and in vivo in the mouse model of chronic carriage. Sci. Rep..

[29] Adhikari S., Khadka S., Sapkota S., Sharma B.R., Ghimire A., Chalise M., Gurung D., Kunwar S. (2020). Multi-drug resistant and extended spectrum β-lactamase producing Salmonella species isolated from fresh chicken liver samples. Kathmandu Univ. Med. J..

[30] Zhang W.-H., Lin X.-Y., Xu L., Gu X.-X., Yang L., Li W., Ren S.-Q., Liu Y.-H., Zeng Z.-L., Jiang H.-X. (2016). CTX-M-27 producing Salmonella enterica serotypes Typhimurium and Indiana are prevalent among food-producing animals in China. Front. Microbiol..

[31] Mthembu T.P., Zishiri O.T., El Zowalaty M.E. (2021). Genomic characterization of antimicrobial resistance in food chain and livestock-associated Salmonella species. Animals.

[32] Forbes Ba W.A., Sahm D.F. (2007).

[33] CLSI (2016).

[34] Chika E., Ifeanyichukwu I., Carissa D., Thomas A., Okoro O., Joshua E., Michael A., Charles E. (2016). Occurrence of metallo-beta-lactamase-producing Enterobacteriaceae from a local poultry farm in abakaliki, Nigeria. Int. J. Appl. Pharm. Sci. Res..

[35] Stepanović S., Vuković D., Dakić I., Savić B., Švabić-Vlahović M. (2000). A modified microtiter-plate test for quantification of staphylococcal biofilm formation. J. Microbiol. Methods.

[36] Kuinkel S., Acharya J., Dhungel B., Adhikari S., Adhikari N., Shrestha U.T., Banjara M.R., Rijal K.R., Ghimire P. (2021). Biofilm Formation and phenotypic detection of ESBL, MBL, KPC and AmpC enzymes and their coexistence in Klebsiella spp. isolated at the national reference laboratory, Kathmandu, Nepal. Microbiol. Res..

[37] Sambrook J., Russell D. (2001).

[38] Varkey D., Veeraraghavan B., Abraham J. (2014). Molecular characterisation of extended spectrum beta lactamase producing strains from blood sample. Int. J. Pharm. Pharmaceut. Sci..

[39] Poirel L., Walsh T.R., Cuvillier V., Nordmann P. (2011). Multiplex PCR for detection of acquired carbapenemase genes. Diagn. Microbiol. Infect. Dis..

[40] Ranjan R., Green S.J., Mason C., Butler D., Vinas N.R., Hsu C.-Y., Wagner N., Thomas W.K., Simpson S., Bivens N., Kehoe P., Tighe S. (2020). Methods to preserve individual bacteria and microbiome samples for nucleic acid analyses without altering cellular structure or integrity. J. Biomol. Tech..

[41] Eromo T., Tassew H., Daka D., Kibru G. (2016). Bacteriological quality of street foods and antimicrobial resistance of isolates in hawassa, Ethiopia. Ethiop. J. Health Sci..

[42] Amare A., Worku T., Ashagirie B., Adugna M., Getaneh A., Dagnew M. (2019). Bacteriological profile, antimicrobial susceptibility patterns of the isolates among street vended foods and hygienic practice of vendors in Gondar town, Northwest Ethiopia: a cross sectional study. BMC Microbiol..

[43] Bizuye A., Tewelde S., Agimas A., Meseret A., Tadele E., Mesfin E. (2014). Bacteriological quality of street vending potato chips in gondar Town,North west ethopia. Int. J. Bacteriol. Virol. Immunol..

[44] Kiranmai B., Kamesh S., Sara S. (2016). A cross-sectional study on microbiological quality of street food in. Int. J. Health Sci. Res..

[45] Islam S., Nasrin N., Rizwan F., Nahar L., Bhowmik A., Ahmed M. (2015). Microbial contamination of street vended. Southeast Asian J. Trop. Med. Publ. Health.

[46] Islam M., Morgan J., Doyle M.P., Phatak S.C., Millner P., Jiang X. (2004). Fate of Salmonella enterica serovar Typhimurium on carrots and radishes grown in fields treated with contaminated manure composts or irrigation water. Appl. Environ. Microbiol..

[47] Gorman R., Bloomfield S., Adley C.C. (2002). A study of cross-contamination of food-borne pathogens in the domestic kitchen in the Republic of Ireland. Int. J. Food Microbiol..

[48] D.O. Cliver, Cutting boards in Salmonella cross-contamination, J. AOAC Int. 89 (n.d.) 538–542. 16640304

[49] P. Amoah, P. Drechsel, R.C. Abaidoo, A. Klutse, Efectiveness of common and improved sanitary washing methods in selected cities of West Africa for the reduction of coliform bacteria and helminth eggs on vegetables, Trop. Med. Int. Health 12 Suppl. 2 (n.d.) 40–50. 10.1111/j.1365-3156.2007.01940.x. 18005314

[50] Ananth M., Rajesh R., Amjith R., Achu A.L., Valamparampil M.J., Harikrishnan M., Resmi M.S., Sreekanth K.B., Sara V., Sethulekshmi S., Prasannakumar V., Deepthi S.K., Jemin A.J., Krishna D.S., Anish T.S., Insija I.S., Nujum Z.T. (2018). Contamination of household open wells in an urban area of trivandrum, Kerala state, India: a spatial analysis of health risk using geographic information system. Environ. Health Insights.

[51] Chakraborty D., Basu S., Das S. (2010). A study on infections caused by Metallo Beta Lactamase producing gram negative bacteria in intensive care unit patients. Am. J. Infect. Dis..

[52] Bashir D., Thokar M.A., Fomda B.A., Bashir G. (2011). Detection of metallo-beta-lactamase (MBL) producing Pseudomonas aeruginosa at a tertiary care hospital in Kashmir. Afr. J. Microbiol. Res..

[53] Nipa M., Mazumdar R., Hasan M., Fakruddin M., Islam S., Bhuiyan H., Iqbal A. (2011). Prevalence of multi drug resistant bacteria on raw salad vegetables sold in major markets of Chittagong city, Bangladesh. Middle East J. Sci. Res..

[54] Nawas T., Mazumdar R., Das S., Nipa M., Islam S., Bhuiyan H., Ahmad I. (2012). Microbiological quality and antibiogram of E. coli, Salmonella and Vibrio of salad and water from restaurants of Chittagong. J Env. Sci Nat Resour..

[55] Raphael E., Wong L., Riley L. (2011). Extended-spectrum beta-lactamase gene sequences in gram-negative saprophytes on retail organic and nonorganic spinach. Appl. Environ. Microbiol..

[56] Valentin L., Sharp H., Hille K., Seibt U., Fischer J., Pfeifer Y., Michael G.B., Nickel S., Schmiedel J., Falgenhauer L., Friese A., Bauerfeind R., Roesler U., Imirzalioglu C., Chakraborty T., Helmuth R., Valenza G., Werner G., Schwarz S., Guerra B., Appel B., Kreienbrock L., Käsbohrer A. (2014). Subgrouping of ESBL-producing Escherichia coli from animal and human sources: an approach to quantify the distribution of ESBL types between different reservoirs. Int. J. Med. Microbiol..

[57] Elshamy A.A., Aboshanab K.M. (2020). A review on bacterial resistance to carbapenems: epidemiology, detection and treatment options. Futur. Sci. OA..

[58] Behzadi P., Urbán E., Gajdács M. (2020). Association between biofilm-production and antibiotic resistance in uropathogenic Escherichia coli (UPEC): an in vitro study. Diseases.

[59] Gurung J., Khyriem A.B., Banik A., Lyngdoh W.V., Choudhury B., Bhattacharyya P. (2013). Association of biofi lm production with multidrug resistance among clinical isolates of Acinetobacter baumannii and Pseudomonas aeruginosa from intensive care unit. Indian J. Crit. Care Med..

[60] Fischer J., Rodríguez I., Baumann B., Guiral E., Beutin L., Schroeter A., Kaesbohrer A., Pfeifer Y., Helmuth R., Guerra B. (2014). blaCTX-M-15-carrying Escherichia coli and Salmonella isolates from livestock and food in Germany. J. Antimicrob. Chemother..

[61] Irrgang A., Tenhagen B.-A., Pauly N., Schmoger S., Kaesbohrer A., Hammerl J.A. (2019). Characterization of VIM-1-producing E. coli isolated from a German fattening pig farm by an improved isolation procedure. Front. Microbiol..

[62] Borowiak M., Szabo I., Baumann B., Junker E., Hammerl J.A., Kaesbohrer A., Malorny B., Fischer J. (2017). VIM-1-producing Salmonella Infantis isolated from swine and minced pork meat in Germany. J. Antimicrob. Chemother..

